# Medical Opinion and Movement

**Published:** 1908-08-29

**Authors:** 


### Medical Opinion and Movement.

OF some interest to medical graduates are the statistics which are issued from time to time regarding the attendance at the various German universities. The latest returns for the summer semester of 1908 show clearly that Munich still attracts by far the largest number of foreign students in medicine, for out of a total of 1,521 students no fewer than 1,018 are foreigners. It must be borne in mind, however, that "foreigners" in these tables are taken to be students who do not belong to the state in which the university exists: thus a student from Berlin would be classed as an "Ausländer" at Munich and *vice versa.* The real percentage of foreign students is very much lower, and the percentage of English-speaking graduate students is by no means high except perhaps at Munich. Berlin comes next with 941 medical students, of whom 324 are foreigners. The highest foreign percentage is, however, furnished by Freiburg, of whose 723 medical students 644 are foreigners. The figures for the other universities are as follows, the numbers in brackets denoting the "Ausländers": Bonn, 316 (37); Breslau, 276 (27); Erlangen, 212 (73); Giessen, 197 (95); Göttingen, 224 (65); Greifswald, 207 (43); Halle, 221 (57); Heidelberg, 479 (389); Jena, 268 (219); Kiel, 381 (131); Königsberg, 243 (59); Leipzig, 499 (257); Marburg, 289 (60); Munster, 138 (2); Rostock, 144 (95); Strassburg, 231 (126); Tübingen, 291 (139); Würzburg, 449 (259). This table is very interesting, as it shows that in many cases the popular university, so far as foreign students is concerned, is not regarded very highly by German medical students, as is most evident in the case of Jena, Heidelberg, and Freiburg. Three universities—those of Berlin, Munich, and Leipzig—seem to be equally popular with foreign and native students, while others, such as Munster, Marburg, and Breslau, do not appear to attract students from outside.

---

SOME interesting experiments carried out by Planche, of Lyons, on a series of 65 nursing mothers, with a view to determining the capacity of women for nursing more than one child at a time, have resulted in conclusions which will go far to get rid of the idea that a woman is incapable of nursing twins without the help of hand-feeding or a second wet-nurse. By weighing their infants before and after each breast feed, he found that women nursing one child normally give 500 to 600 grammes of milk during the first month, and that this amount is gradually increased until at the end of twelve months the production of milk is 1,200 grammes. If a second child be put to the breast after the first has fed, in course of time the secretion is doubled. If a third child be suckled after the second has finished, a still greater increase is gradually induced, so much so that in one case as much as 2,600 grammes of milk were given. Thus this hyper-secretion is the result of a purely mechanical process, the suction of the nipple. No drug is capable of augmenting this secretion; the hyper-secretion so produced is not the result of a proliferation of the gland tissue, for the breasts of the women experimented on, showed no marked increase in size. Planche explains this fact by supposing that normally only a certain number of the acini of a nursing breast are called upon to function, and that the increased excitation of the nipple, due to the supplementary nurslings, calls the inactive acini into play. He further remarks that the nurslings in no way suffered by being nursed by the same woman, but rather the reverse, their appetites increasing along with their weights.

---

THE frequent use of calomel as an internal remedy lends a particular interest to the question of its manner and place of absorption. M. Nemzere (*Roussky Vratch*), at the Institute of Experimental Medicine of St. Petersburg, has undertaken researches into this question. His experiments were conducted upon dogs which had been subjected to artificial fistulæ at various points of the gastro-intestinal tract. The author finds that during its stay in the digestive tube the calomel undergoes a process of solution, solubility, however, varying in different places. In the stomach little or none is absorbed, despite the presence of free hydrochloric acid; solubility increases much in the neighbourhood of the duodenum where the reaction is still acid, and reaches its maximum in the small intestine. Mercury, which reaches the large intestine in solution, is there partly absorbed, the other part being precipitated by the sulphuretted hydrogen. Bile and gastric juice have absolutely no action upon the mercury, which is only acted upon by the succus entericus and, more especially, by the pancreatic juice. The alkaline reaction of the intestinal content does not prevent the process of absorption of calomel, which starts in the small intestine and stops, according to all appearances, in the upper segments of the large bowel. A fairly large portion of the mercury absorbed is stored for a relatively long time in the liver, the kidneys, and the thickness of the walls of the large intestine—that is to say, precisely in those organs upon which calomel exerts a selective action in stimulating their functions. Other viscera retain but little calomel, and behave indifferently with regard to it.

---

THE advances in surgical technique during the last decade have been chiefly directed towards aseptic and mechanical measures of sterilisation and away from the use of chemicals. Still, the latter are deemed indispensable in this country for certain purposes, particularly the preparation of the site of operation, and of the surgeon's hands and gloves. Dr. Dukeman, however, a Californian surgeon, describes in the *Medical Record* his routine system which dispenses with the use of antiseptics altogether, and he claims to secure results which compare favourably with those of all other methods. His patients are cleansed by scrubbing with sterile gauze sponges, sterile soft soap, and sterile water at 120° F.; he avoids the use of brushes as unnecessary and irritating. After that the skin is washed with equal parts of alcohol and ether, and the field of operation is covered up with a thin poultice of sterile soft soap for one to three hours; then the poultice is removed, and the washing process repeated, after which a compress of sterile gauze is applied and bandaged on. After anæsthetisation, the soap treatment is once more repeated on the operating table; then the skin is well rinsed, sponged with ether and then with alcohol, and finally irrigated with hot sterile saline solution. The disinfection of the patient's mouth is not neglected, and even his nose is douched with one per cent. salt solution. This last seems somewhat drastic, but Dr. Dukeman's motto is evidently that of Wentworth, for he orders "several" examinations of the urine lest no pathological condition of the kidney be overlooked.

THE surgeon's technique of preparation is somewhat similar. Gloves are worn only when dealing with septic cases, and only then to avoid fouling the hands; the attendant nurses, however, always wear them. The preparation of the hands commences overnight with soap and water and a boiled nail-brush; but the latter is not used on the back of the hands, nor on the arms. In the washing room the nostrils are thoroughly cleansed, and a sterile operating suit, including gauze headgear, is donned. After more washing, the hands and arms are immersed in hot saline solution contained in a deep receptacle. Then they are washed with alcohol, and again rinsed in the sterile saline. The first incision is made by one stroke of a knife through the skin and subcutaneous fat; this instrument is then discarded, and a new one used for the deep structures. Artery forceps are hardly ever used, bleeding being controlled with hot sterile compresses. Even retractors are avoided as far as may be, so as to bruise the tissues as little as possible. Arteries are tied before division, whenever they can be detected; no ligatures larger than No. 2 are used, and they are to be tied snugly, but not too tightly, a somewhat vague expression which might mean anything. In after-treatment, the most conspicuous feature is the unusually low diet which Dr. Dukeman believes in. He allows one or two ounces of hot broth every three hours after twelve to twenty-four hours of starvation following the operation. Aperients are ordered on the third or fourth day, and nothing but liquid is allowed until the bowels have been open. Even then only very light and semi-liquid food is allowed up to the end of a week. Inasmuch as the regimen before operation is practically starvation for three days, it is certainly wonderful that collapse is encountered as seldom as the author asserts. Dr. Dukeman is evidently a surgeon with convictions, and the courage of them.

THE exact relation of optic neuritis to intracranial tumours is analysed very thoroughly by Mr. Paton in a very instructive paper read before the Ophthalmological Society, based upon the consideration of no fewer than 252 cases. In temporo-sphenoidal and in cerebellar tumours this symptom is universal, and hæmorrhages are quite frequent. With tumours of the frontal and parietal lobes papillitis is almost always found, but with subcortical tumours only in about 60 per cent., and in pontine cases is rather fewer still. He confirms previous observers in asserting that the nature of the growth does not influence in any way consequent neuritis. As to intensity of the swelling, Mr. Paton thinks that no diagnostic evidence is afforded by comparison of the two discs, either from the quantity of the swelling or of the priority of its onset. This conclusion is, of course, at variance with that usually held and taught by neurologists. The only deduction which the author will definitely commit himself to on this question of intensity is this: that in cortical tumours the intensity of the neuritis varies inversely with the distance of the part affected from the anterior pole of the middle fossa. As to the nature of the process, so much debated, which produces the appearance and symptoms of optic neuritis, his conclusions are mainly negative. From post-mortem evidence it is held that inflammation does not descend by the meninges. The frequent association of unimpaired vision with great papillitis is against the descent of inflammation along the nerve trunk. Pressure in the nerve sheath is believed to be equally out of court; neither is there evidence of œdema due to toxines, nor of vasomotor disturbances.

A GOOD deal of research work has recently been expended on the metabolism of the chlorides in the body, which goes to show that they are an important factor in certain pathological conditions, in regard to the quantity injected and absorbed into the system or in regard to their elimination and excretion by the kidneys. Reference has already been made in these columns to the relation between hyperchlorination and polyuria. Dr. L. A. Gluzinski propounds the view, as the result of certain observations, that in the course of renal arterio-sclerosis and chronic interstitial nephritis, periods of chlorine retention occur, accompanied by general symptoms, sometimes of a serious nature; such are chiefly vomiting and severe thirst. There are, however, no muscular pains, headache, or mental symptoms. Gluzinski designates the condition under the name of "a-chlorinated uræmia," to distinguish it from ordinary uræmia. The condition may terminate favourably in the course of a few days, or it may end fatally with symptoms of extreme weakness and loss of consciousness, but without convulsions. In acute nephritis, also, the author is of opinion that there is a preliminary stage, which precedes the albuminuria and the other recognised classical symptoms of the disease, during which there is a retention of chlorides in the system giving rise to a characteristic group of symptoms: weakness, vomiting, slight œdema, especially of the face, and diminution in the quantity of urine. In this way, by repeated analyses of the urine during convalescence from scarlatina the author claims that the onset of post-scarlatinal nephritis may be diagnosed before the disease has definitely developed.

THE value of the operation of uretero-cystoneostomy or cysto-ureteral anastomosis has been called in question, and the fear expressed that subsequent cicatricial contraction of the new orifice would impede the flow of urine. Dr. G. Rouscher has recently reported upon thirteen cases in which the operation has been carried out, and as some of these have been examined three years after the operation, the results recorded are of considerable value. Two separate methods have been followed in these cases. In the one the ureter was maintained in position by a suture carried out through the urethra; in the other the extremity of the ureter was split, and each lip was fixed to the bladder wall by means of two sutures perforating the bladder from within outwards as far as possible from the position of the new urethral orifice. The first method yielded five cures, one stenosis, and the result of a seventh case is unknown. The second method gave also five cures and one stenosis. So that, according to these figures, the two methods appear of equal value. Five cases were carried out by the extraperitoneal route with excellent results, but the remaining eight operations were performed by the transperitoneal route, and of these three resulted in stenosis. Consideration must, however, be given to the condition under which the operation takes place. The anastomosis is effected in a region formed essentially of connective tissues, and is especially liable, therefore, to infection. In infective cases, therefore, healing by first intention is likely to fail, and cicatricial contraction follows in consequence. Nevertheless, the successes reported show that in spite of these unfavourable conditions good results can be obtained. The time at which stenosis is manifested varies from five to eight months after operation.

---

ALTHOUGH massage of the heart is a recognised method of treatment for sudden arrest of the heart either in the course of anæsthesia or from severe hæmorrhage, cases of recovery are sufficiently rare to be worthy of record. Dr. A. Van Zanten, a Dutch physician, reports an interesting case of this nature. He received an urgent call to attend a woman who had just been delivered of a child at full term, and who had been taken with a severe post-partum hæmorrhage which the midwife in attendance was unable to control. He found the patient unconscious and bathed in a pool of blood. A rapid examination showed that the hæmorrhage was due to an extensive laceration of the cervix. Plugging arrested the hæmorrhage, but the patient was *in extremis*. Injections of camphor, etc., were of little avail, and after a few minutes the pulse became so bad that it could only be slightly felt from time to time. Dr. Van Zanten then had recourse to massage of the heart, following a rhythm of about seventy pressures a minute. A short time later the beat of the heart could no longer be detected. Ten minutes later, however, the pulse again became perceptible, beating about 120. As soon, however, as the operator ceased his manipulations the pulse became imperceptible again. These changes recurred again twice, when fatigue necessitated a pause in the manipulations. Finally, however, the pulse became stronger, the cyanosis diminished, respiration improved, and the patient recovered consciousness. Massage was kept up altogether for an hour and a quarter. The patient eventually recovered completely. The fact that the action of the heart varied so uniformly in response to the manipulations points to the importance of the massage in the eventual recovery of the patient, and is evidence of the efficacy of the treatment in such extreme conditions. No doubt much depends upon its adoption at a sufficiently early stage.

---

LIGATURE of the portal vein is recognised as incompatible with the continuation of existence, and so it undoubtedly is under ordinary circumstances, but Ito and Omi have shown that it can be tolerated when a collateral circulation has been previously provided by preliminary omental fixation, by the fistula of Eck, and by successive and superposed ligatures of the branches of the vessel. Drs. Brewer and Gies, of New York, have recently reported a case which appears to demonstrate also that the main portal vessel can be ligatured with impunity when by the pressure of a tumour upon it a collateral circulation has been sufficiently developed. The case was one of hydatid cyst of the left lobe of the liver. An incision was made in the epigastrium, and a large oval tumour presented, pushing forward the gastro-hepatic mesentery and the stomach. The mesentery was divided in the middle line, and there appeared then a glistening, greyish tissue, which was supposed to be the fibrous wall of the cyst. An exploring needle was introduced and clear fluid withdrawn. On withdrawing the needle, however, there was a profuse hæmorrhage from the puncture. It was then discovered that what had been taken for the cystic wall was in reality a large vein passing from the pancreatic region to the interior of the liver. The vein was flattened and stretched by the tumour. Attempts to suture failed, and finally two ligatures were applied above and below the tear. The cyst was then opened and emptied and left to drain. The patient recovered and subsequently showed no symptoms suggestive of interference with the portal system, and yet there seems little doubt that the vein ligatured was actually the main portal trunk. The explanation of this immunity appears to be that the growth of the tumour and consequent pressure on the main vessel had gradually caused a deviation of the portal circulation into collateral and accessory paths, which were eventually sufficient to carry on the entire portal circulation. It was noted in the course of the abdominal incision that there was a well-marked development of the subcutaneous vessels around the umbilicus, such as is observed in cases of cirrhosis of the liver.

---

*The next issue of "The Hospital" will be the Annual EDUCATIONAL NUMBER, and will contain leading articles on Medical Education in the United Kingdom, together with detailed information concerning the various medical schools, examining bodies and post-graduate institutions, and special articles on the Public Services.*

